# Supplementary material for: Dietary Calcium and Protein Levels Influence Growth Performance, Intestinal Development, and Nutrient Utilization in Goslings
Source: Vet Sci. 2025 Mar 28;12(4):310. doi: 10.3390/vetsci12040310 (PMC12031493; doi:10.3390/vetsci12040310)
Supplement: Supplementary file 1 [file vetsci-12-00310-s001.zip › vetsci-3516942-supplementary.pdf]

Table S1. Effect of calcium × protein levels on growth performance of goslings (two way ANOVA).

Item	Ca (%)	Protein (%)	BW of 1 day (g)	BW of 30 day (g)	ADFI (g)	ADG (g)	FCR
LCLP	0.32	14.5	99.12	1825.87	114.96	57.56	2.00
LCMP	0.32	18.5	99.35	1777.92	114.74	55.95	2.05
LCHP	0.32	22.5	99.44	1734.86	115.97	54.51	2.14
MCLP	0.96	14.5	99.54	1839.52	128.25	58.00	2.21
MCMP	0.96	18.5	99.17	1809.92	130.71	57.03	2.29
MCHP	0.96	22.5	99.26	1639.10	111.34	54.49	2.06
HCLP	2.88	14.5	99.31	914.58	74.49	26.62	2.82
HCMP	2.88	18.5	99.35	849.33	60.57	24.83	2.45
HCHP	2.88	22.5	99.07	706.81	56.62	20.26	2.80
P-value	Ca × CP		0.423	0.660	0.039	0.922	0.015

Table S2. Effect of calcium × protein levels on serum UA, Cr and UN of goslings (μmol/L) (two way ANOVA).

Item	Ca (%)	Protein (%)	14d			30d		
			UA	Cr	UN	UA	Cr	UN
LCLP	0.32	14.5	180.20	33.67	3.13	242.17	33.83	4.33
LCMP	0.32	18.5	200.20	33.50	2.32	271.33	40.67	1.33
LCHP	0.32	22.5	230.83	43.55	3.80	318.17	44.83	3.37
MCLP	0.96	14.5	229.90	17.77	0.77	230.35	17.53	0.79
MCMP	0.96	18.5	314.58	22.28	0.49	255.90	13.50	0.83
MCHP	0.96	22.5	278.43	21.35	0.66	262.3	19.10	1.62
HCLP	2.88	14.5	313.80	38.80	3.43	238.83	26.50	3.35
HCMP	2.88	18.5	329.40	54.62	2.05	246.00	35.83	2.92
HCHP	2.88	22.5	283.00	54.95	3.35	324.67	30.33	2.38
P-value	Ca × CP		0.004	<0.001	<0.001	0.121	<0.001	<0.001

Table S3. Effect of calcium × protein levels on serum Ca and P of goslings (μmol/L) (two way ANOVA).

Item	Ca (%)	Protein (%)	14d		30d	
			Ca	P	Ca	P
LCLP	0.32	14.5	2.59	2.50	2.52	2.47
LCMP	0.32	18.5	2.53	2.54	2.54	2.43
LCHP	0.32	22.5	2.60	2.53	2.55	2.37

MCLP	0.96	14.5	2.35	2.38	2.44	2.17
MCMP	0.96	18.5	2.29	2.39	2.46	2.18
MCHP	0.96	22.5	2.25	2.39	2.52	2.03
HCLP	2.88	14.5	2.34	2.27	2.64	2.48
HCMP	2.88	18.5	2.35	2.37	2.49	2.47
HCHP	2.88	22.5	2.54	2.57	2.58	2.30
P-value	Ca × CP		0.246	0.282	0.227	0.919

Table S4. Effects of calcium × protein levels on duodenal morphology of goslings aged 14 and 30 days (two way ANOVA).

Item	Ca (%)	Protein (%)	14d		30d	
			Villus height	Crypt depth	Villus height	Crypt depth
LCLP	0.32	14.5	811.07	226.93	936.68	245.92
LCMP	0.32	18.5	889.76	210.26	1011.94	228.60
LCHP	0.32	22.5	854.86	209.50	1114.13	254.12
MCLP	0.96	14.5	1008.90	235.08	1130.63	241.21
MCMP	0.96	18.5	1029.40	226.93	1030.76	235.37
MCHP	0.96	22.5	926.91	230.08	1088.18	237.08
HCLP	2.88	14.5	665.52	176.98	796.24	254.70
HCMP	2.88	18.5	819.57	202.77	740.53	209.75
HCHP	2.88	22.5	745.67	225.34	714.24	211.38
P-value	Ca × CP		0.303	0.127	0.210	0.029

Table S5. Effects of calcium × protein levels on jejunal morphology of goslings aged 14 and 30 days(μm) (two way ANOVA).

Item	Ca (%)	Protein (%)	14d		30d	
			Villus height	Crypt depth	Villus height	Crypt depth
LCLP	0.32	14.5	1025.01	246.68	887.70	203.21
LCMP	0.32	18.5	1063.23	243.79	1162.88	242.84
LCHP	0.32	22.5	1077.12	238.44	1254.11	273.25
MCLP	0.96	14.5	977.56	260.63	1349.45	252.23
MCMP	0.96	18.5	818.18	210.96	1223.79	250.90
MCHP	0.96	22.5	875.33	252.07	1299.01	249.52
HCLP	2.88	14.5	702.24	215.15	1030.26	211.76
HCMP	2.88	18.5	861.37	216.44	1049.18	194.17
HCHP	2.88	22.5	879.61	199.63	1207.22	227.33
P-value	Ca × CP		0.062	0.295	0.025	0.006

Table S6. Effects of calcium × protein levels on ileal morphology of goslings aged 14 and 30 days (two way ANOVA).

Item	Ca (%)	Protein (%)	14d		30d	
			Villus height	Crypt	Villus height	Crypt depth

			depth			
LCLP	0.32	14.5	830.45	218.84	922.82	203.35
LCMP	0.32	18.5	848.30	259.51	968.42	262.71
LCHP	0.32	22.5	899.20	246.03	844.65	225.26
MCLP	0.96	14.5	734.80	243.82	968.90	217.85
MCMP	0.96	18.5	657.29	228.38	1207.13	222.11
MCHP	0.96	22.5	730.97	231.99	1027.05	229.84
HCLP	2.88	14.5	545.84	218.06	841.33	204.39
HCMP	2.88	18.5	798.60	238.51	813.50	192.11
HCHP	2.88	22.5	764.45	237.68	834.64	229.58
P-value	Ca × CP		0.001	0.280	0.039	0.006

Table S7. Effects of calcium × protein on duodenal digestive enzyme activities of 14 and 30-day old goslings (U/mg prot) (two way ANOVA).

Item	Ca (%)	Protein (%)	14d			30d		
			α-AMS	LPS	TPS	α-AMS	LPS	TPS
LCLP	0.32	14.5	0.19	14.33	162.91	0.88	9.45	146.04
LCMP	0.32	18.5	0.15	19.07	222.46	0.99	13.12	152.68
LCHP	0.32	22.5	0.17	24.00	233.80	0.77	11.38	124.58
MCLP	0.96	14.5	0.24	32.59	219.09	0.56	9.69	131.09
MCMP	0.96	18.5	0.26	25.77	207.82	0.57	9.98	105.80
MCHP	0.96	22.5	0.20	25.06	199.50	0.51	9.30	124.42
HCLP	2.88	14.5	0.46	31.03	177.19	0.68	13.85	111.31
HCMP	2.88	18.5	0.34	32.15	200.78	0.88	13.47	138.87
HCHP	2.88	22.5	0.21	19.13	192.47	0.84	13.74	115.92
P-value	Ca × CP		<0.001	<0.001	0.030	0.001	0.023	0.001

Table S8. Effects of calcium × protein on jejunal digestive enzyme activities of 14 and 30-day old goslings (U/mg prot) (two way ANOVA).

Item	Ca (%)	Protein (%)	14d			30d		
			α-AMS	LPS	TPS	α-AMS	LPS	TPS
LCLP	0.32	14.5	0.21	46.23	202.69	0.54	14.91	176.41
LCMP	0.32	18.5	0.17	50.89	185.52	0.56	9.36	148.73
LCHP	0.32	22.5	0.20	54.21	175.51	0.64	14.29	222.91
MCLP	0.96	14.5	0.35	53.97	126.00	0.43	12.06	203.75
MCMP	0.96	18.5	0.27	46.60	145.53	0.52	7.12	133.44
MCHP	0.96	22.5	0.29	61.12	110.73	0.72	16.02	234.50
HCLP	2.88	14.5	0.28	61.90	97.61	0.60	12.75	189.85
HCMP	2.88	18.5	0.31	69.33	75.32	0.70	7.09	151.97
HCHP	2.88	22.5	0.35	64.23	92.41	0.66	14.53	144.07
P-value	Ca × CP		<0.001	0.002	0.029	<0.001	0.001	<0.001

Table S9. Effects of calcium × protein on ileal digestive enzyme activities of 14 and 30-day old goslings (U/mg prot) (two way ANOVA).

Item	Ca (%)	Protein (%)	14d			30d		
			α-AMS	LPS	TPS	α-AMS	LPS	TPS
LCLP	0.32	14.5	0.36	19.00	202.76	0.53	4.82	156.52
LCMP	0.32	18.5	0.35	26.95	231.25	0.33	3.77	133.93
LCHP	0.32	22.5	0.36	24.37	297.02	0.37	4.41	154.15
MCLP	0.96	14.5	0.27	26.26	259.40	0.55	3.31	204.15
MCMP	0.96	18.5	0.36	25.26	256.38	0.74	4.28	147.86
MCHP	0.96	22.5	0.30	35.88	260.08	0.42	4.25	139.42
HCLP	2.88	14.5	0.36	26.10	133.22	0.29	4.63	166.00
HCMP	2.88	18.5	0.27	27.43	168.99	0.20	3.40	142.89
HCHP	2.88	22.5	0.32	14.75	235.80	0.22	4.83	125.28
P-value	Ca × CP		<0.001	<0.001	0.016	<0.001	<0.001	0.005

Table S10. Effect of calcium × protein on the utilization of calcium, phosphorus and protein in goslings (two way ANOVA).

Item	Ca (%)	Protein (%)	Apparent metabolic rate (%)		
			Ca	P	Protein
LCLP	0.32	14.5	39.94	45.15	65.04
LCMP	0.32	18.5	39.45	41.32	65.05
LCHP	0.32	22.5	36.53	39.82	62.32
MCLP	0.96	14.5	56.51	43.39	64.26
MCMP	0.96	18.5	58.07	48.51	66.29
MCHP	0.96	22.5	52.33	46.61	61.60
HCLP	2.88	14.5	65.25	39.35	73.79
HCMP	2.88	18.5	63.36	36.49	57.09
HCHP	2.88	22.5	63.95	40.39	63.65
P-value	Ca × CP		0.024	0.001	0.004
